# Location-Specific Spectral and Thermal Effects in Tracking and Fixed Tilt Photovoltaic Systems

**DOI:** 10.1016/j.isci.2020.101634

**Published:** 2020-10-01

**Authors:** José M. Ripalda, Daniel Chemisana, José M. Llorens, Iván García

**Affiliations:** 1 Instituto de Micro y Nanotecnología - CSIC, Isaac Newton, 8, E-28760, Tres Cantos, Madrid, Spain; 2 Applied Physics Section of the Environmental Sci. Department, Universitat de Lleida, Jaume II 69, 25001 Lleida, Spain; 3 Instituto de Energía Solar, Universidad Politécnica de Madrid, Avda. Complutense 30, 28040, Madrid, Spain

**Keywords:** Energy Resources, Energy Management, Energy Systems

## Abstract

The efficiency of photovoltaic modules in the field is generally lower than the efficiency under standard testing conditions due to temperature and spectral effects. Using the latest spectral dataset available from the National Solar Radiation Database, we report spectral correction factors ranging from -2% to 1.3% of the produced energy for silicon modules depending on location and collector geometry. We find that spectral effects favor trackers if silicon modules are used, but favor a fixed tilt instead if perovskites or CdTe are used. In high-irradiance locations, the energy yield advantage of silicon-based trackers is underestimated by 0.4% if spectral sensitivity effects are neglected. As the photovoltaic market grows to a multi-terawatt size, these seemingly small effects are expected to have an economic impact equivalent to tens of billions of dollars in the next few decades, far outweighting the cost of the required research effort.

## Highlights

• The Si gap is lower than optimal, but less so when the sun is near the horizon
• Spectral effects favor trackers if silicon modules are used
• For perovskites and CdTe, a fixed tilt is favored by spectral effects

## Graphical Abstract

**Figure:**
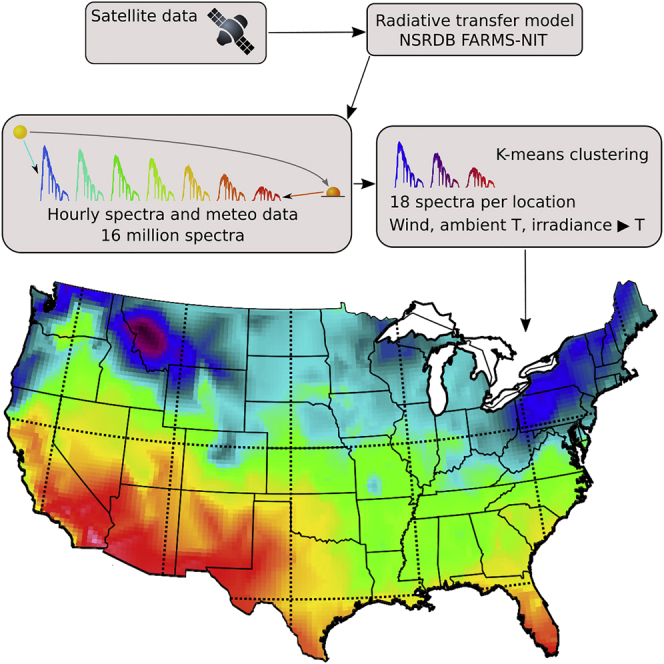

## Introduction

Due to the rapid cost reduction of photovoltaics (PV), recent forecasts are predicting that several tens of terawatts of PV capacity will be deployed before 2050 (Haegel et al., 2019). This represents an investment of several tens of trillions of dollars. But the actual performance of PV systems in the field is highly variable depending on a number of factors (Krauter and Hanitsch, 1996; Krauter, 2006), and as a consequence there is a large economic drive to optimize the choice of location and technology for new PV systems. Key aspects to take into account are the geographical and temporal variations of the spectral irradiance and meteorological parameters such as ambient temperature and wind speed. Changes in the spectral irradiance are mostly driven by the position of the sun and atmospheric conditions (Kurtz et al., 1991; Chan et al., 2014; Ripalda et al., 2018; Vossier et al., 2017; Garcia et al., 2018; Dirnberger et al., 2015; Fernández et al., 2014; Huld, 2017; Kinsey, 2015; Huld and Gracia-Amillo, 2015; Lindsay et al., 2020; Peters et al., 2018; Warmann and Atwater, 2019), and also by the orientation of solar panels as defined by the plane of array (POA). Accurately accounting for these effects requires detailed radiative transfer models including multiple reflection, scattering, and absorption events in the atmosphere including both cloudy and clear-sky conditions (Xie and Sengupta, 2018; Xie et al., 2019b). Data from these radiative transfer models has only recently become widely available through the National Solar Radiation Database (NSRDB) for North American locations (Xie and Sengupta, 2018; Xie et al., 2019b; Sengupta et al., 2018). Here we use these spectral and meteorological datasets to obtain the PV efficiency and energy production as a function of location for a wide range of PV technologies with the United States as a case study. We include the effects of wind, ambient temperature, and irradiance on solar cell temperatures in addition to spectral variability and the effects of clouds. We also compare spectral effects in tracking systems with fixed tilt systems. Our results exemplify that consideration of the combined effects of spectral and temperature variations will allow to fine-tune the optimal location, module technology, and collection geometry for each PV project, with an economic benefit far outweighting the cost of the required research effort. Most importantly, we provide spectral correction factors, for each location and PV technology, that can be used to improve the accuracy of conventional energy production forecasts.

In the first section of this work we are concerned with the implications of thermal and spectral variability for mainstream PV technology based on fixed tilt silicon modules. Thin film technologies such as CdTe and perovskites are also discussed. We then examine the implications of our study for the energy production of tracking systems. In the next section we also consider multijunctions under global spectra. We then quantify the band gap adjustments required at specific locations to maximize the produced energy. To conclude we discuss the uncertainty in our results as a function of the number of spectra used per location.

**Figure 1:**
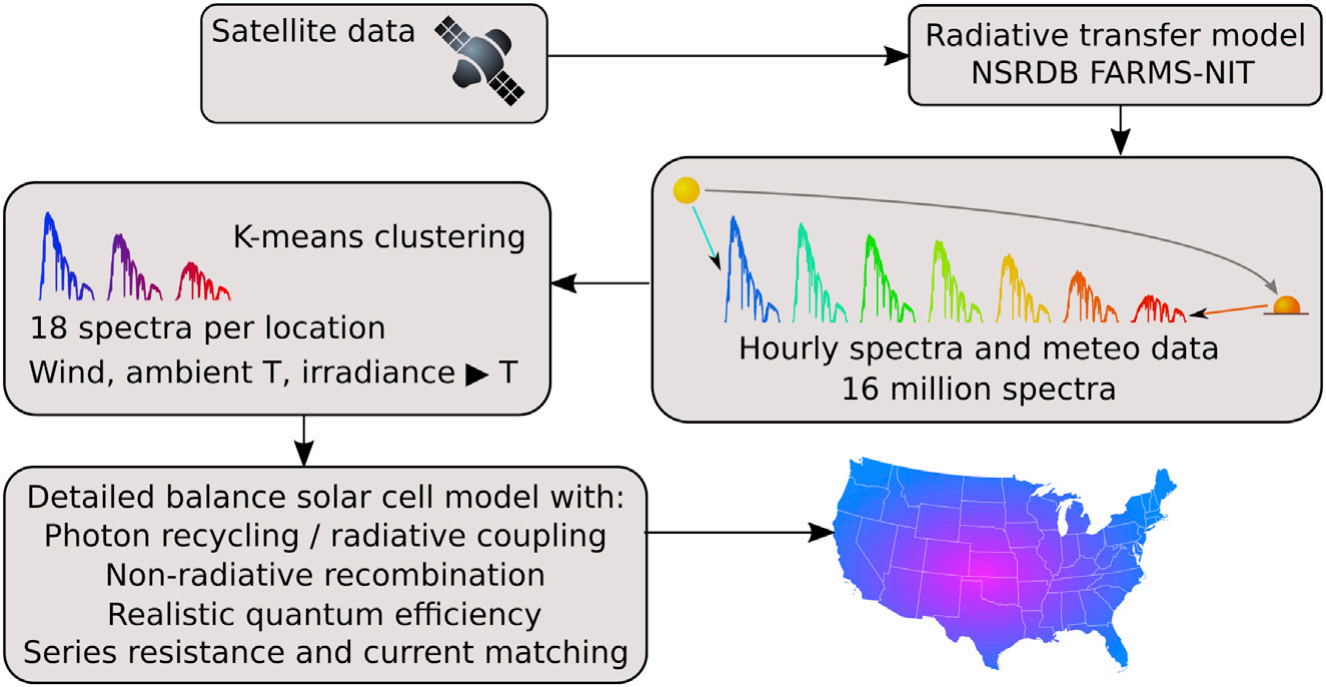
Flow Chart of the Methodology to Calculate the Yearly Energy Yield and Time-Averaged PV Efficiency as a Function of Location Further details are given in the Methods section.

A flow chart summarizing our methodology is shown in Figure 1. We have included in our calculations the most relevant effects as detailed in the Experimental Procedures and in Ripalda et al. (2018). We use the Sandia PV Array Performance Model for solar cell temperatures using ambient temperature and wind speed data (King et al., 2004). In single junctions, the most pronounced effect of temperature is a reduction in the voltage due to a higher recombination current (Dupré et al., 2015, 2017). An appropriate model for solar cell temperatures is also required because of the Varshni shift of the band gaps with temperature.

## Results

**Figure 2:**
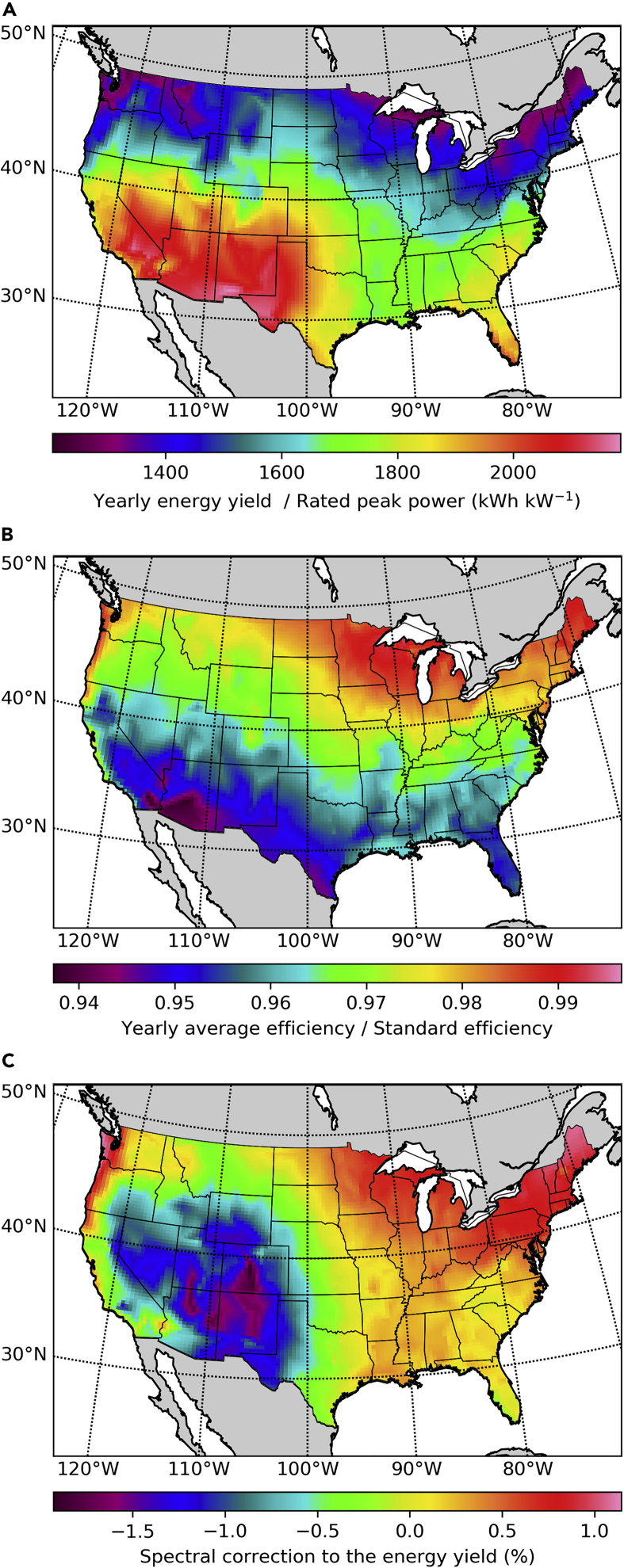
Silicon Single Junctions at a Fixed Optimal Tilt Angle (A) Yearly energy production relative to the rated peak power of the PV system. (B) Yearly averaged efficiency relative to the standard efficiency. (C) Spectral correction factors to be used when calculating the efficiency or the energy production assuming the standard spectrum.

A typical PV system uses silicon modules fixed at a tilt angle roughly matching the latitude and oriented toward the south (or to the north if in the southern hemisphere). We adjust the tilt angle for each location as a function of latitude according to the prescription given by Jacobson and Jadhav (2018). We have calculated the maximum realistically achievable yearly energy production (Figure 2A) and yearly averaged energy efficiency (Figure 2B) for such systems when considering spectral and temperature variability effects. The trends in the energy production map are opposite to those in the efficiency map due to the effect of higher solar cell temperatures in high-irradiance locations. The lower efficiency in the south is mostly due to the effect of temperature on the recombination current, and consequently on the voltage, but high temperatures also further shift the silicon band gap away from the optimal value for a single junction (1.35 eV according to Ripalda et al., 2018). Temperature effects slightly reduce the economic advantage of deployment in high-irradiance locations.

To quantify spectral sensitivity effects we compute spectral correction factors $f_{s}$ as the ratio of the yearly energy yield $E_{\text{y}}$ obtained with the NSRDB POA spectral irradiance s(λ) and the yearly energy yield obtained assuming the standard ASTM G-173-03 spectrum $s_{0}\left(\lambda \right)$ scaled to match the NSRDB POA irradiance $G=\int_{0}^{\infty }s\left(\lambda \right)\;d\lambda$ as

(Equation 1) $$f_{s}=\frac{E_{\text{y}}\left(s\left(\lambda \right)\right)}{E_{\text{y}}\left(s_{0}\left(\lambda \right)\;G/G_{0}\right)},$$

where $G_{0}$ is the integrated irradiance of the standard spectrum. To clearly separate spectral effects, the spectra are the only difference between these two energy yield calculations. Because the standard solar spectrum is often assumed to forecast the expected energy yield of new PV power plants, these spectral correction factors can be used to correct such forecasts. But these spectral correction factors can also be used to illustrate the relative importance of spectral sensitivity effects for each location and type of PV system. We present in Figure 2C the resulting spectral correction percentage as $\left(f_{s}-1\right)$. Neglecting spectral effects thus leads to overestimating the energy yield in some of the locations with the highest production potential by nearly 2% (high-altitude locations in Colorado and New Mexico), whereas slightly underestimating it in others (the Sonoran Desert at the border between California and Arizona).

Spectral variability effects in single junctions are due to the absorption threshold of the semiconductor. These effects show a clear correlation between topographic altitude and efficiency losses in Figure 2C. By comparing the spectra at low-altitude locations with the spectra from locations at high altitude, we observe that the efficiency is the highest at low altitude due to higher infrared losses caused mostly by the water content of the atmosphere. Because these losses occur at energies below the band gap, they have the effect of an apparent efficiency increase that is not necessarily accompanied by an energy yield increase.

### CdTe and Perovskites

**Figure 3:**
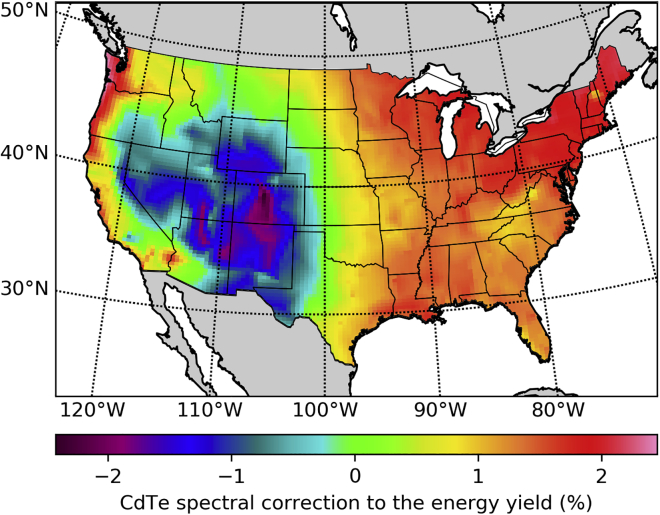
CdTe Yearly Spectral Correction Factor to the Energy Yield and the Efficiency

As a consequence of the rapid drop in price of silicon-based PV modules with higher efficiencies, the market share of thin film PV technologies based on CdTe and CuGaInSe_2_ has declined slightly in recent years, but thin film technologies are likely to maintain a foothold in certain markets, applications, or geographical regions. Here we center our attention on the case of CdTe, as its higher band gap (1.45 eV for CdTe versus 1.12 eV for silicon) might make it advantageous in locations with lower infrared irradiance or higher temperatures. The spectral correction factors in Figure 3 do indeed show a wide geographical variation range.

In comparison with silicon, the performance of CdTe modules is more dependent on spectral effects due to its higher band gap. As recently suggested by Peters et al. (2018), this is related to infrared losses in the atmosphere caused by water, decreasing the POA irradiance without decreasing the energy yield as these changes occur at energies below the CdTe band gap. As a consequence the efficiency of CdTe and perovskite single junctions increases with increasing precipitable water in the atmosphere.

**Figure 4:**
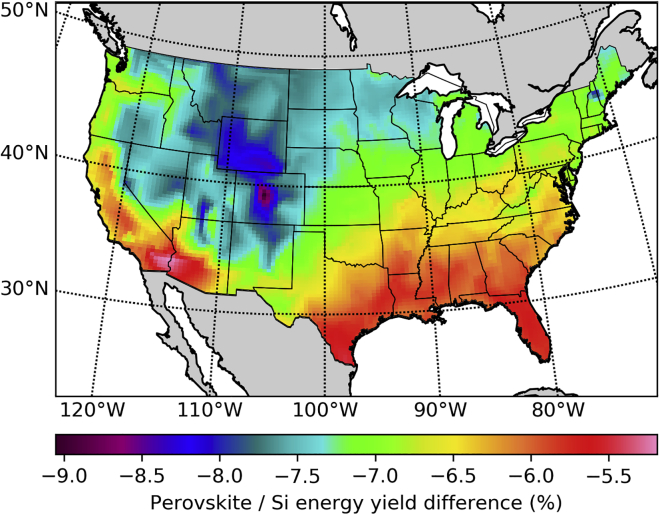
Relative Energy Yield Difference between Perovskite Single Junctions and Silicon (Perovskite-Si/Si)

Due to their similar band gap, the geographical distributions of the energy yield, the efficiency, and the spectral correction factor are very nearly the same for CdTe and perovskites, but with lower non-radiative recombination losses favoring perovskites over CdTe as we have optimistically assumed the performance parameters of record perovskite solar cells before degradation (Jung et al., 2019). Further details are given in the Methods section. To compare the yearly energy yield of perovskite modules $E_{\text{y}}^{\text{P}}$ with the energy yield of silicon $E_{\text{y}}^{\text{Si}}$ in a fixed optimal tilt geometry, we plot in Figure 4 the relative energy yield difference between perovskites and silicon as $E_{\text{y}}^{\text{P}}/E_{\text{y}}^{\text{Si}}-1$. The POA irradiance is the same in both cases because the collection geometry is the same, and consequently the efficiency ratio is the same as the energy yield ratio (this will not be the case when studying the effect of tracking). We have assumed here the band gap of the current record solar cell (1.5 eV) (Jung et al., 2019). The relevant result in Figure 4 is the relative difference between locations, and not the absolute result at each location, as the actual performance of perovskite modules in the field is still largely unknown and we have not included time-dependent degradation effects (Tress et al., 2019).

### Tracking

**Figure 5:**
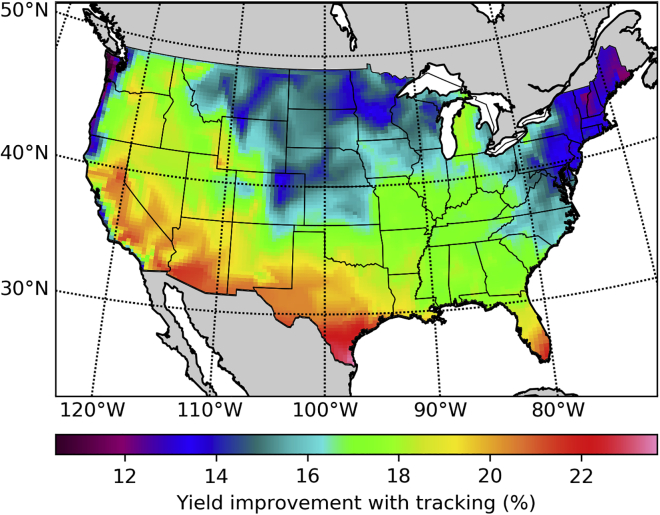
Yearly Energy Yield Improvement Factor Obtained by Mounting Silicon PV Modules on Horizontal Single Axis Trackers Rather than at a Fixed Tilt Angle

Among all the utility-scale PV systems installed in the United States in 2016, 80% were tracking systems (Fu et al., 2017). The most common type of PV tracking is currently horizontal single axis tracking (HSAT). We present in Figure 5 the ratio of the yearly energy production of silicon-based HSAT systems relative to that of fixed tilt systems.

**Figure 6:**
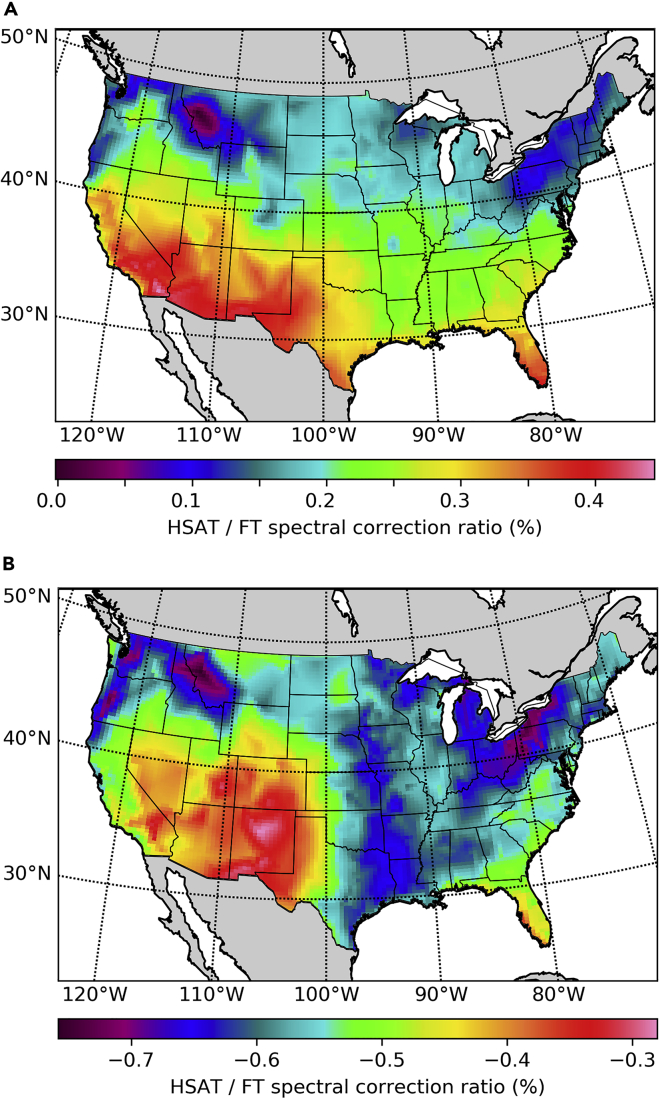
Ratio of Spectral Correction Factors for Tracking (HSAT) and Fixed Tilt (FT) Systems (A) Silicon. (B) Perovskite.

Using tracking to increase the average irradiance on the POA comes at the cost of increased solar cell temperatures. But thermal effects are only slightly detrimental to the efficiency of HSAT systems because their higher yearly energy production (Figure 5) is mostly due to a more spread out energy production along the course of each day, and not to significantly higher peak POA irradiances. Furthermore, we find that the spectral correction factors for silicon-based tracking systems are more favorable than those of fixed tilt systems. The ratio of spectral correction factors for HSAT ($f_{s}^{\text{HSAT}}$) and fixed tilt systems ($f_{s}^{\text{FT}}$) is presented as a percentage as $\left(f_{s}^{\text{HSAT}}/f_{s}^{\text{FT}}-1\right)$ in Figure 6. For silicon systems (Figure 6A), tracking is found to be favored by spectral effects in all the locations that we have studied, and comparing Figure 2A with Figure 6A reveals that the spectral correction ratio is most favorable for tracking systems in those areas with the highest yearly energy production. This is related to the efficiency peak due to spectral matching during the early and late hours of the day described by Krauter and Hanitsch (1996). Spectral effects further increase the energy yield advantage of silicon-based trackers because trackers collect more sunlight during sunrise and sunset, and during these times the spectrum peaks at lower energies due to the higher air mass. Furthermore, tracking also increases the direct fraction of the plane of array irradiance, further lowering the average photon energy. Although this increases losses due to photons with energy lower than the band gap, the effect that prevails is a reduction in carrier thermalization losses, as the band gap of silicon (1.12 eV) is smaller than the optimal band gap for maximum yearly energy production (1.35 eV) (Ripalda et al., 2018). Conversely, if perovskites or other high band-gap single junctions are used, spectral effects favor a fixed tilt geometry, as shown in Figure 6B. Because the POA irradiance of tracking systems is higher than the POA irradiance of fixed tilt systems, the energy yield is always higher for trackers, but this advantage is reduced in the case of perovskite absorbers due to spectral effects.

Spectral effects cause an apparently small energy yield difference of about 0.4% between trackers and fixed tilt systems, but this difference has important techno-economic consequences as it directly affects the net profit margin of PV power plants, which is typically not much larger than a few percent. The capital cost of utility-scale PV power plants is around 1 $/W and decreasing at a 4% yearly rate (Fu et al., 2017; ITRPV, 2020, 2020b). The capital cost difference between fixed tilt and tracker systems is only 7.7%, and operating costs are 15.4 $/kW-y and 18.5 $/kW-y, respectively. Thus the magnitude of spectral effects is also sizable in comparison with the incremental cost of tracking systems.

### Multijunctions

**Figure 7:**
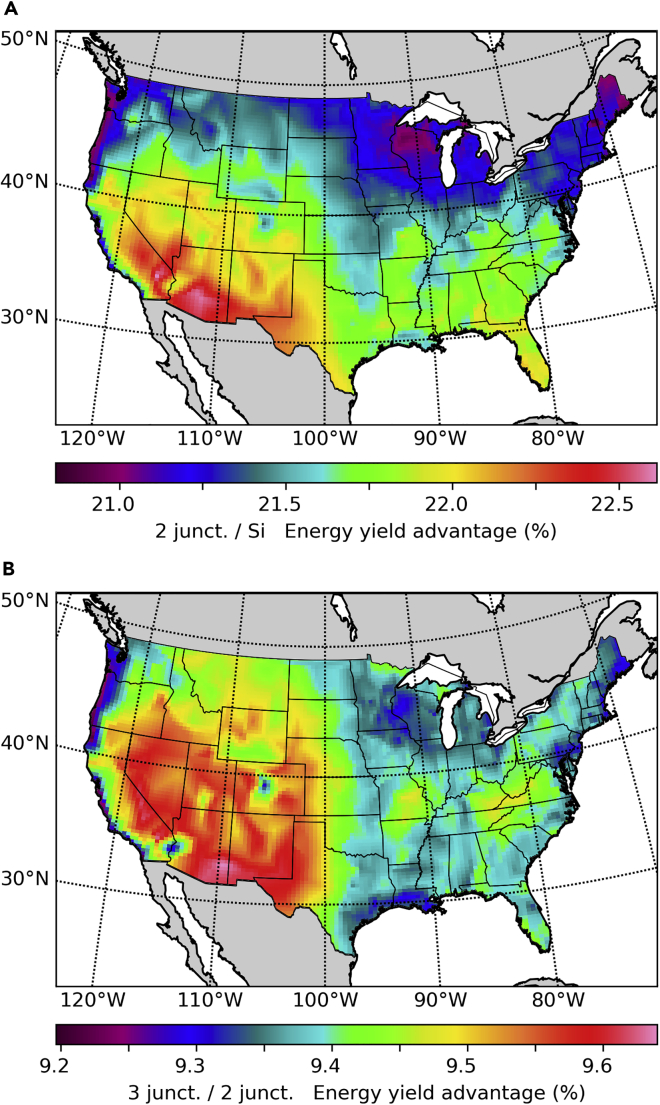
Energy Yield Ratios as a Function of the Number of Junctions The POA irradiance is here the same in all cases, so the efficiency ratio is the same as the energy yield ratio. (A) Ratio of the energy yield obtained with an optimal double junction to the energy yield of silicon under global irradiance with horizontal single-axis tracking. (B) Ratio of the energy yield obtained with an optimal triple junction to the energy yield of an optimal double junction under global irradiance with horizontal single-axis tracking.

An often raised concern about multijunction technology is its sensitivity to spectral variations. We present in Figure 7A the energy yield ratio of an optimal series connected double junction relative to a silicon single junction under global irradiance with HSAT. In this case the POA irradiance is the same for both systems, and thus the energy yield ratio is the same as the yearly average efficiency ratio. The band gaps of the dual junction here discussed are those found as optimal in our previous work, 1.126 eV and 1.687 eV for the bottom and top junctions, respectively (Ripalda et al., 2018).

We find that double junctions are most advantageous in high-irradiance locations due to a lower sensitivity to high temperatures. In the south, a 22% yearly energy yield advantage might provide a market entrance opportunity for dual junction modules, especially in the residential market, where area constraints increase the value of high-efficiency systems, and modules only represent about 10% of the total cost (Fu et al., 2017), allowing for multijunction module costs three times higher than current module costs. This target might be compatible with a recent cost reduction road map for III-V multijunctions published by NREL (Essig et al., 2017). Alternatively, multijunctions based on perovskites have recently surpassed the efficiency of silicon single junctions (Green et al., 2020), and reported degradation rates are also improving rapidly (Hou et al., 2020; Xu et al., 2020).

The areas most favorable for multijunctions in Figure 7A have a large overlap with the regions most favorable for tracking in Figure 7B. This reinforces a synergy between these two technologies given by the fact that the revenue generated by a PV system results from the product of a number of factors such as solar cell efficiency, inverter efficiency, cell interconnection efficiency, POA irradiance, and transmission of antireflective coatings and encapsulating materials. An increase in any of these factors makes it more profitable to invest in increasing any of the other factors.

The technical complexity and cost of multijunctions has a super-linear trend with the number of junctions, whereas the efficiency has a sub-linear increase with the number of junctions. So it remains unclear what would be the number of junctions that maximizes the return on investment, partly due to location-dependent effects. The relative improvement in the yearly energy production obtained by replacing an optimal double junction with a triple junction is shown in Figure 7B. In both cases the bottom junction is chosen to be silicon, as its band gap is nearly optimal, and it has a high performance/cost ratio. The middle and top junction band gaps for the triple junction are those found as optimal for a silicon-based series connected triple junction in our previous work, 1.48 and 1.94 eV, respectively (Ripalda et al., 2018).

As expected, the spectral correction factors for the series connected double and triple junctions (not shown) are more adverse than those of the silicon single junction. They follow a geographical pattern closely matching that in Figure 2C, suggesting that the spectral sensitivity effects are mostly given by the absorption threshold of the silicon bottom junction. The spectral corrections range from −3.4% to −1.1% for the double junction and −4.4% to −2.1% for the triple junction. If the photocurrent from the silicon bottom junction is collected separately using a three- or four-terminal configuration (neglecting cell interconnection losses), the spectral corrections range from −2.2% to 1.5% for the double junction and −4.0% to −0.7% for the triple junction. So a multi-terminal configuration is most beneficial for the double junction. The energy yield of multi-terminal silicon-based tandems using technologically relevant but not optimal band gaps (GaAs and GaInP) has recently been studied by several groups (Schulte-Huxel et al., 2018; Essig et al., 2017; Liu et al., 2020). Using the corresponding band gaps (1.42 eV for GaAs and 1.85 eV for GaInP) with our model, we reproduce the results reported by Schulte-Huxel et al., (2018), obtaining larger gains for the multi-terminal configuration than when using optimal band gaps. Thus the multi-terminal configuration is of most interest when the optimal band gaps for the series connected configuration cannot be used due to technological constraints. This conclusion is also supported by the recent work by Mathews et al. (2020). A multiterminal configuration is also of interest for bifacial silicon-based tandems, as the yearly energy yield advantage of bifacial cells is otherwise limited by current matching constraints (Onno et al., 2020).

**Figure 8:**
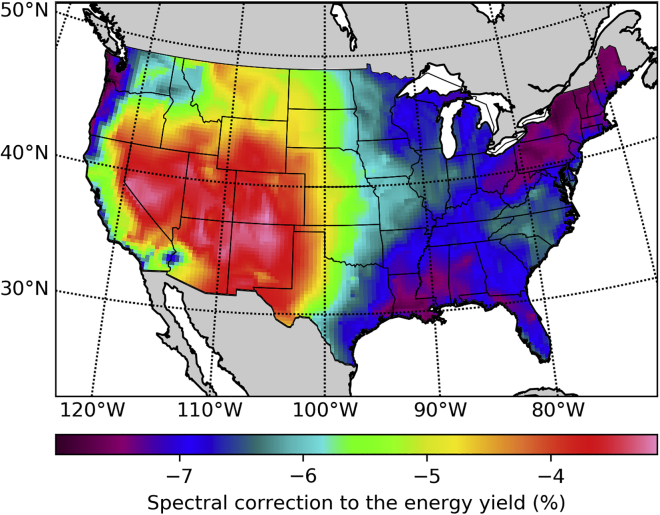
Spectral Correction Factor for the Yearly Energy Production of a Six-Junction Solar Cell with the Band Gaps of the Current Record Device under Global Irradiance (Geisz et al., 2018).

As an example of extreme spectral sensitivity, we have considered the case of an optimal series connected six-junction device under global spectra. The band gaps of the six-junction architecture here discussed are those of the current record for a solar cell under the global spectrum (Geisz et al., 2018; Green et al., 2020). The energy yield advantage over silicon single junctions ($E_{\text{y}}^{6\text{j}}/E_{\text{y}}^{\text{Si}}-1$) ranges from 50.8% in the Rocky Mountains to 38.7% in New England. The spectral correction factor for the six-junction device is shown in Figure 8. The geographical pattern is almost the opposite of all the previous cases, with high-altitude locations being the least adversely affected by spectral sensitivity effects. This different pattern here suggests that the spectral sensitivity of this device is of a fundamentally different type than in the previous cases. Silicon and silicon-based multijunctions have a spectral sensitivity mostly determined by the absorption threshold of silicon, whereas the spectral sensitivity of this six-junction device is mostly given by the current matching constraint. High altitude reduces losses caused by the atmosphere, reducing spectral variability and current mismatch effects in the six-junction case.

### Fine-Tuning for Specific Locations

As recently discussed by Parent et al., the energy yield of multijunctions can be increased by optimizing the band gaps using local spectra and meteorological conditions (Parent et al., 2019). We have re-optimized the band gaps of silicon-based triple junctions at a few representative locations. In the data presented here (Table 1) the bottom junction band gap is fixed as we assume a silicon-based tandem. If this constrain is relaxed, we find the required bottom junction band gap adjustments to reach a local efficiency maximum are typically small, whereas top junctions are the ones that require larger adjustments. This is expected because, regardless of geographical location, the efficiency maxima as a function of the bottom junction band gap are mostly given by the atmospheric absorption band thresholds, as discussed by McMahon et al. (2017). Higher top junction band gaps are favored in hot areas as this leads to a reduction of recombination voltage losses, but spectral effects lead to exceptions to this rule, as in the case of the North Pacific coast, where high band gaps are favored due to spectral effects. The obtained efficiency improvement is typically of about 0.5%.

**Table 1:** Fine-Tuning of Band Gaps at Specific Locations for Series Constrained Silicon-Based Triple Junctions

	Mid. Gap	Top Gap	Ref. Eff.	Eff.
	eV	eV	%	%
Leadville, CO	1.490	1.975	34.95	35.37
Denver, CO	1.494	1.978	34.81	35.23
Mojave, CA	1.499	1.981	34.70	35.26
Tucson, AZ	1.500	1.986	34.33	34.95
Astoria, OR	1.504	1.987	35.40	36.16

The reference efficiency is obtained with middle and top junction band gaps of 1.48 and 1.94 eV, respectively.

### Uncertainty versus Number of Spectra

Our results suggest that forecasting the yearly energy production of PV systems requires location-specific solar spectra. Yearly spectral sets with thousands of spectra per year and location are available from the NSRDB (Sengupta et al., 2018; Xie and Sengupta, 2018; Xie et al., 2019b). The dataset used in this work comprises 16 million spectra, each with 2002 wavelengths and associated meteorological data. The number of required spectra can be reduced using statistical techniques such as binning (Garcia et al., 2018), and machine learning clustering (Ripalda et al., 2018). Here we cluster the spectra not only according to their spectral content as in our previous work (Ripalda et al., 2018) but also according to other correlated meteorological data such as wind speed and ambient temperature, as these also have an effect on PV efficiency.

In the previous sections we have used 18 clustered spectra per location, as we have previously determined that this leads to an uncertainty in the results typically smaller than 0.2% while still reducing the computational cost by several orders of magnitude (Ripalda et al., 2018).

**Figure 9:**
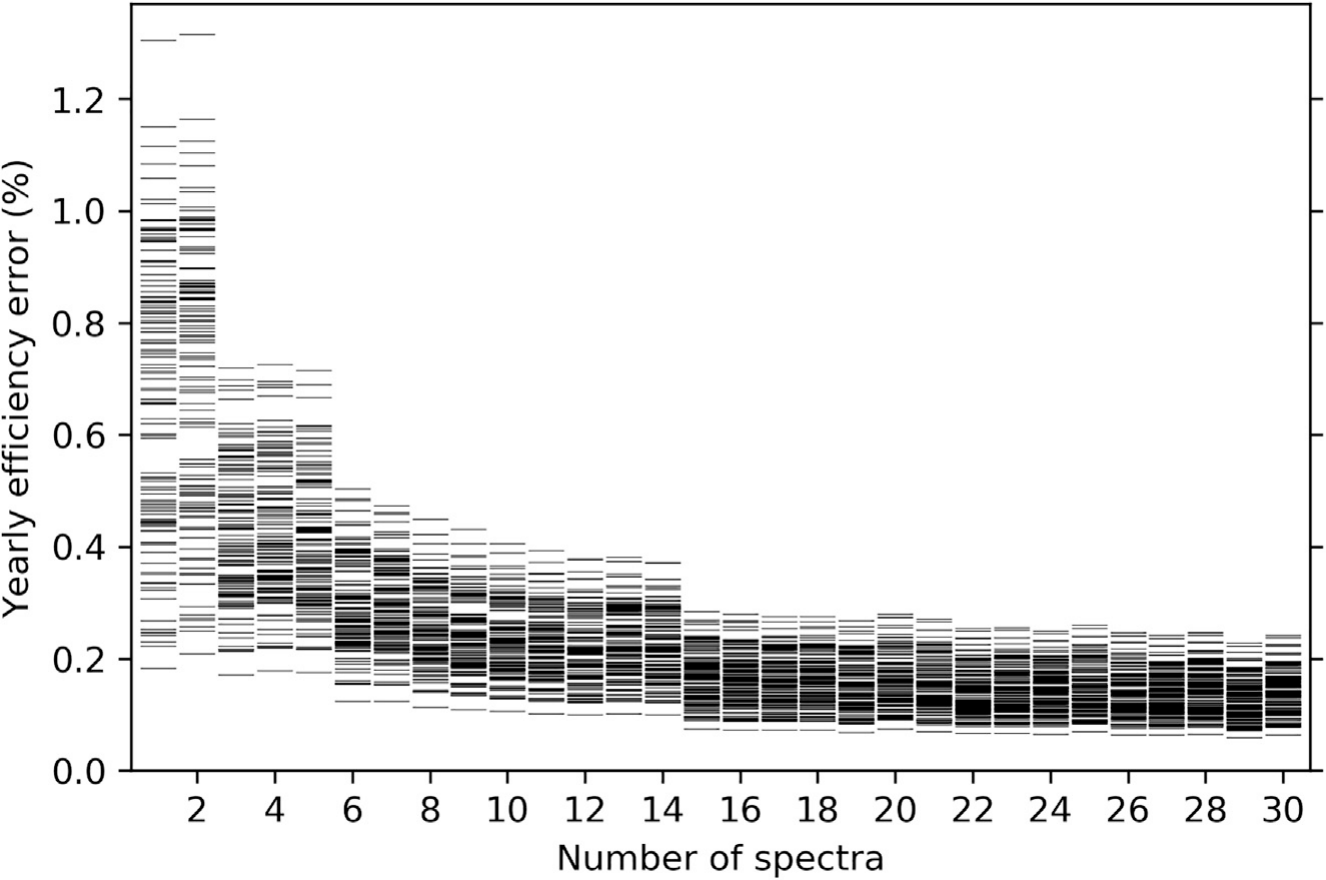
Convergence of the Yearly Averaged Efficiency as a Function of the Number of Clustered Spectra This example corresponds to a set of 140 triple junctions with random but nearly optimal band gaps (efficiency within 2% of the maximum) on a horizontal single-axis tracker at Elko, Nevada.

In this section we study how the quality of the obtained results improves as the number of spectra is increased, using as a reference the results obtained with the whole dataset. In Figure 9 we show the efficiency error statistics as a function of the number of spectra for triple junction modules on HSAT. As shown in Figure 9, there is little benefit obtained by increasing the number of proxy spectra beyond 20, and the uncertainty in energy yield forecasts is likely to be dominated by other factors such as the uncertainties on the spectrally integrated irradiance, module degradation, soiling rates, and other loss mechanisms at the module and system level, which are out of the scope of this work.

## Discussion

The contiguous United States spans a wide range of latitudes and atmospheric conditions. As a consequence, the yearly averaged PV efficiency of silicon modules can vary with location by up to 1.4% absolute efficiency. Spectral sensitivity effects account for about half of the geographical variability of efficiencies, with spectral correction factors ranging from −2.0% to 1.1% in terms of the energy yield, or −0.5%–0.3% in terms of absolute efficiency. We find that thermal effects predominate over spectral sensitivity, and slightly reduce the economic advantage of high irradiance locations. The former are mostly determined by latitude and irradiance, whereas the latter are mostly determined by topographical altitude and atmospheric phenomena. Spectral sensitivity effects are found to favor silicon HSAT over fixed tilt systems by up to 0.45% of the energy yield in high-irradiance locations at lower latitudes, but a fixed tilt geometry is favored instead for perovskites and other high band-gap absorbers such as CdTe. The energy yield loss caused by spectral sensitivity in multijunctions is found to be roughly proportional to the number of junctions. If the optimal band gaps are used, this loss is not significantly mitigated by using a multi-terminal configuration for current extraction. Nevertheless, because the choice of band gaps is often constrained, multi-terminal configurations greatly enhance the flexibility of multijunction design. The data here presented clearly show that location-specific solar spectra are required for accurate predictions of the energy yield, but rather than sets of thousands of spectra for each location covering a whole year, it suffices to use a few characteristic spectra for each location. A possible future line of research, inspired by the recent work by Warmann and Atwater (2019) is to identify a set of geographical, atmospheric, and meteorological parameters that correctly predicts the yearly energy yield in all locations within an acceptable uncertainty margin. However, the most pressing need at the moment seems to be to decrease the uncertainty margin in the spectral irradiance data used as input in models such as the one here discussed. Such advances should lead to significantly reduced uncertainty in PV production forecasts, and consequently lower risk and financial cost for PV projects.

### Limitations of the Study

Although out of the scope of the present work, in practice there are other important effects on the energy production and return on investment of PV installations, such as the higher degradation rate with increased module temperatures (Ascencio-Vásquez et al., 2019; Vazquez et al., 2017) and the geographical and seasonal variations of soiling rates (García et al., 2011; Sarver et al., 2013; Ilse et al., 2019), as well as other loss mechanisms at the module and system levels. When PV module temperatures are above a certain threshold, it becomes economically advantageous to turn trackers away from the sun, reducing the risk of damage and also the energy production. Similarly, the frequency of cleaning is also determined by a balance between the energy yield and operating costs. Both effects are specially adverse in arid regions with high temperatures and infrequent rain. We reduce the reported specific energy yields by 3% due to shadow losses, 2% due to inverter clipping and inverter efficiency losses, 2% due to system degradation and failures, 2% due to soiling, and 1% due to other effects such as DC and AC wire losses and mismatch losses, among others. We do not attempt to do a location-dependent bottom-up model of system level losses, as these effects have little correlation with the spectral effects, which are the main subject of this work.

Ground-based observations of the solar spectra are available from a limited number of locations, covering a limited time span. Therefore a consistent large dataset covering large regions and time spans can only be obtained from modeled data based on satellite images, and later validated by comparison with spectra measured from the ground (Sengupta et al., 2015). The NREL-NSRDB spectra used in this work have been derived from the FARMS-NIT model and validated in comparison with measured spectra from the NREL Solar Radiation Research Laboratory in Xie et al. (2019a). These ground-based measurements unavoidably have their own uncertainties and biases, and thus complete agreement with physics-based models cannot be expected. The FARMS-NIT data in comparison with surface-based observations have shown a percentage error in the 1.86% to 2.28% range, whereas the previous model from NREL (TMYSPEC) had percentage errors ranging from −3.47% to −16.27% (Xie et al., 2019a). Although this represents a large improvement over the previous state of the art, there is still a clear need to reduce these uncertainties to improve the bankability of PV systems. The potential economic return of such advances clearly justifies the required research effort. In the meantime, the qualitative trends here illustrate phenomena that need to be accounted for to improve PV energy production forecasts.

The spectral irradiance data for HSAT systems provided by the NSRDB is obtained in the limit of low ground cover ratios without back-tracking (Xie et al., 2019a). Systems with high ground cover ratios can be expected to be less sensitive to the changes in the spectra during the early morning and late afternoon.

### Resource Availability

#### Lead Contact

Dr. José María Ripalda Cobián, email: j.ripalda@csic.es.

#### Materials Availability

No materials or samples where handled in this research.

#### Data and Code Availability

The data and code required to reproduce the results here presented are available as open source at https://github.com/Ripalda/Tandems.

## Methods

All methods can be found in the accompanying Transparent Methods supplemental file.

## Supplemental Information

Document S1. Transparent Methods
