# Supplementary material for: Location-Specific Spectral and Thermal Effects in Tracking and Fixed Tilt Photovoltaic Systems
Source: iScience. 2020 Oct 1;23(10):101634. doi: 10.1016/j.isci.2020.101634 (PMC7569229; doi:10.1016/j.isci.2020.101634)
Supplement: Document S1. Transparent Methods [file mmc1.pdf]

iScience, Volume *23*

Supplemental Information

Location-Specific Spectral and Thermal Effects in Tracking and Fixed Tilt Photovoltaic Systems

José M. Ripalda, Daniel Chemisana, José M. Llorens, and Iván García

SUPPLEMENTARY MATERIAL

Transparent Methods

Solar cell model and proxy spectra. We have used a modified detailed balance method with a set of proxy spectra obtained from clustering of yearly spectral sets as described in Ripalda, Buencuerpo, and García, 2018. Building on state of the art understanding of fundamental losses (Hirst and Ekins-Daukes, 2011), our model is based on previous work by Geisz *et al.* and García *et al.*. (Geisz et al., 2015; Garcia et al., 2018). All the parameters in the model are the same as in our previous work with the exception of the external radiative efficiency for CdTe, where we optimistically assumed 0.01% rather than the default value of 1%. A parameter sensitivity analysis including all relevant parameters in our model can be found in Ripalda, Buencuerpo, and García, 2019. The global spectral irradiance data was downloaded from the National Solar Radiation Database (NSRDB) as derived from the FARMS-NIT model valid for all sky conditions, including the effects of clouds (Xie and Sengupta, 2018; Xie, Sengupta, and Wang, 2019). The used spectra and meteorological data are historical data from 2017. The spectral irradiance is modified by an angle of incidence modifier accounting for increased reflectance at grazing angles. We have used the physically based angle of incidence modifier implemented in the PVLIB python open source library (F. Holmgren, W. Hansen, and A. Mikofski, 2018; De Soto, Klein, and Beckman, 2006).

The initial data set includes 16 million spectra (8760 spectra for 913 locations and 2 different collection geometries). These are reduced to 18 characteristic spectra per location used as a proxy for the whole yearly data-set. The proxy spectra are obtained by machine learning clustering of the initial data set (Ripalda, Buencuerpo, and García, 2018). The band gaps were optimized for maximum yearly energy yield as described in ibid.

Temperature model. We obtain solar cell temperatures from the ambient temperature, the irradiance, and the wind speed using the PVLIB python open source library implementation of the empirically fitted Sandia photovoltaic array performance model using the default parameters for a polymer-back open rack array (F. Holmgren, W. Hansen, and A. Mikofski, 2018). To include the effect of efficiency on solar cell temperatures, the irradiance values used as input in the Sandia temperature model are modified by a thermal correction factor that is a function of the efficiency, as the efficiency of the modules used to fit the Sandia temperature model was rather low ($\eta_0 = 16.5\%$), and the power carried away as electricity does not contribute to raise the solar cell temperature. This factor is given by $(1 - R - \eta) / (1 - R - \eta_0)$, where η is the efficiency, and R is the reflectivity. The efficiency values used to calculate the thermal correction factor are those in Table 1 of Ripalda, Buencuerpo, and García, 2018 to avoid the need for self consistent iteration, as the temperature would depend on the efficiency and vice versa. The value assumed for R is arbitrarily chosen to be 0.05, but the sensitivity of the results to both R or η is small (doubling the value assumed for R or increasing the efficiency by 5% reduces the resulting temperature by 0.5°C and this increases the efficiency of a silicon single junction by 0.03%).

References

De Soto, W., S.A. Klein, and W.A. Beckman (2006). "Improvement and validation of a model for photovoltaic array performance". In: Solar Energy 80.1, pp. 78–88. ISSN: 0038092X. DOI: 10.1016/j.solener.2005.06.010. URL: https://linkinghub.elsevier.com/retrieve/pii/S0038092X05002410 (visited on 01/31/2020).

F. Holmgren, William, Clifford W. Hansen, and Mark A. Mikofski (2018). "pvlib python: a python package for modeling solar energy systems". In: Journal of Open Source Software 3.29, p. 884. ISSN: 2475-9066. DOI: 10.21105/joss.00884. URL: http://joss.theoj.org/papers/10.21105/joss.00884 (visited on 01/10/2020).

Garcia, Iván et al. (2018). "Spectral binning for energy production calculations and multijunction solar cell design". en. In: Progress in Photovoltaics: Research and Applications 26.1, pp. 48–54. ISSN: 10627995. DOI: 10.1002/pip.2943. (Visited on 08/22/2018).

Geisz, John F. et al. (2015). "Generalized Optoelectronic Model of Series-Connected Multijunction Solar Cells". In: IEEE Journal of Photovoltaics 5.6, pp. 1827–1839. ISSN: 2156-3381, 2156-3403. DOI: 10.1109/JPHOTOV.2015.2478072. (Visited on 06/06/2017).

Hirst, Louise C. and Nicholas J. Ekins-Daukes (2011). "Fundamental losses in solar cells". en. In: Progress in Photovoltaics: Research and Applications 19.3, pp. 286–293. ISSN: 10627995. DOI: 10.1002/pip.1024. URL: http://doi.wiley.com/10.1002/pip.1024 (visited on 03/19/2020).

Ripalda, J. M., J. Buencuerpo, and I. García (2018). "Solar cell designs by maximizing energy production based on machine learning clustering of spectral variations". en. In: Nature Communications 9.1, p. 5126. ISSN: 2041-1723. DOI: 10.1038/s41467-018-07431-3. URL: http://www.nature.com/articles/s41467-018-07431-3 (visited on 04/22/2019).

Ripalda, J. M., J. Buencuerpo, and I. García (2019). "Dependence of Multijunction Optimal Gaps on Spectral Variability and Other Environmental and Device Parameters". In: IEEE PVSC 46 Proceedings, Chicago.

Xie, Yu and Manajit Sengupta (2018). "A Fast All-sky Radiation Model for Solar applications with Narrowband Irradiances on Tilted surfaces (FARMS-NIT): Part I. The clear-sky model". In: Solar Energy 174, pp. 691–702. ISSN: 0038092X. DOI: 10.1016/j.solener.2018.09.056. URL: https://linkinghub.elsevier.com/retrieve/pii/S0038092X18309502 (visited on 01/21/2020).

Xie, Yu, Manajit Sengupta, and Chenxi Wang (2019). "A Fast All-sky Radiation Model for Solar applications with Narrowband Irradiances on Tilted surfaces (FARMS-NIT): Part II. The cloudy-sky model". In: Solar Energy 188, pp. 799–812. ISSN: 0038092X. DOI: 10.1016/j.solener.2019.06.058. URL: https://linkinghub.elsevier.com/retrieve/pii/S0038092X19306334 (visited on 01/17/2020).
